# Supplementary material for: Endothelial EphB4 maintains vascular integrity and transport function in adult heart
Source: eLife. 2019 Nov 29;8:e45863. doi: 10.7554/eLife.45863 (PMC6884395; doi:10.7554/eLife.45863)
Supplement: Supplementary file 1. — List of all the materials and resources used. [file elife-45863-supp1.docx]

| **Key Resources Table** | | | | |
| --- | --- | --- | --- | --- |
| **Reagent type (species) or resource** | **Designation** | **Source or reference** | **Identifiers** | **Additional information** |
| strain, strain background (*Mus musculus*, C57BL/6JRj) | WT | Janvier Labs |  |  |
| genetic reagent (*Mus musculus*) | *EphB4*^flox^ | Wang et al., 2015 |  |  |
| genetic reagent (*Mus musculus*) | *Efnb2*^flox^ | Grunwald et al., 2004 |  |  |
| genetic reagent (*Mus musculus*) | *Cdh5-CreERT2* | Wang et al., 2010 |  |  |
| genetic reagent (*Mus musculus*) | *Prox1-CreERT2* | Bazigou et al., 2011 |  |  |
| genetic reagent (*Mus musculus*) | *Rosa26RmTmG* | Muzumdar et al., 2007 |  |  |
| genetic reagent (*Mus musculus*) | *Efnb2::GFP* | Davy and Soriano, 2007 |  |  |
| cell line (*Homo-sapiens*) | Human Umbilical Vein Endothelial Cells (HUVEC) | ThermoFisher | Cat# C0035C |  |
| transfected construct (include species here) | Negative control siRNA | Ambion | Cat# 4390844 |  |
| transfected construct (include species here) | *EPHB4* siRNA | Ambion | Cat# 4390824 |  |
| transfected construct (include species here) | *CAV1* siRNA | ThermoFisher | Cat# HSS141466 |  |
| antibody | anti-IB4-Biotin (Griffonia Simplicifolia) | Vector | Cat# B-1205 RRID:AB_2314661 | IF (1:25) |
| antibody | anti-GFP (Chicken, polyclonal) | 2BScientific | Cat# GFP-1010 RRID:AB_2307313 | IF (1:100) |
| antibody | WGA-tetramethylrhodamine | Invitrogen | Cat# W849 | IF (1:100) |
| antibody | anti-EphB4 (Rat, monoclonal) | Hycult Biotechnology | Cat# HM1099 RRID:AB_10346396 | IF (1:100) |
| antibody | anti-Ephb4 (Goat, polyclonal) | R&D Systems | Cat# AF446 RRID:AB_2100105 | IF (1:100)  WB (1:2000) |
| antibody | anti-Icam2 (Rat, monoclonal) | BD Pharmingen | Cat# 553326 RRID:AB_394784 | IF (1:100) |
| antibody | anti-SMA-Cy3 (Mouse, monoclonal) | Sigma | Cat# C6198 RRID:AB_476856 | IF (1:100) |
| antibody | anti-PdgfrB (Rat, monoclonal) | eBioscience | Cat# 14-1402-82 RRID:AB_467493 | IF (1:100) |
| antibody | anti-Ter-119 (Rat, monoclonal) | R&D Systems | Cat# MAB1125 RRID:AB_2297123 | IF (1:100) |
| antibody | anti-Vegfr3 (Rabbit, polyclonal) | ReliaTech | Cat# 102-PA22S | IF (1:50) |
| antibody | anti-Collagen Type I (Rabbit, polyclonal) | Millipore | Cat# AB765P RRID:AB_92259 | IF (1:100) |
| antibody | anti-Collagen Type IV (Goat, polyclonal) | Millipore | Cat# AB769 RRID:AB_306025 | IF (1:100) |
| antibody | anti-ERG (Rabbit, monoclonal) | Abcam | Cat# ab110639 RRID:AB_10864794 | IF (1:100) |
| antibody | anti-CD31 (Goat, polyclonal) | R&D Systems | Cat# AF3628 RRID:AB_2161028 | IF (1:100) |
| antibody | anti-GAPDH (Rabbit, monoclonal) | Cell Signaling | Cat# 2118 RRID:AB_561053 | WB (1:1000) |
| antibody | anti-phospho-Akt (Ser473) (Rabbit, monoclonal) | Cell Signaling | Cat# 4060 RRID:AB_2315049 | WB (1:1000) |
| antibody | anti-Akt (Rabbit, monoclonal) | Cell Signaling | Cat# 4691 RRID:AB_915783 | WB (1:1000) |
| antibody | anti-p44/42 MAPK (Rabbit, monoclonal) | Cell Signaling | Cat# 4695 RRID:AB_390779 | WB (1:1000) |
| antibody | anti-phospho-p44/42 (The202/Thr204) (Rabbit, monoclonal) | Cell Signaling | Cat# 4370 RRID:AB_2315112 | WB (1:1000) |
| antibody | anti-Cav-1 (Rabbit, monoclonal) | Cell Signaling | Cat# 3238 RRID:AB_637792 | IF (1:100) WB (1:50000) |
| antibody | anti-beta-Actin (Mouse, monoclonal) | Santa Cruz | Cat# sc-47778 RRID:AB_2714189 | WB (1:6000) |
| antibody | anti-Src (Rabbit, monoclonal) | Cell Signaling | Cat# 2123 RRID:AB_2106047 | WB (1:1000)  IP (1:50) |
| antibody | anti-phospho-Cav-1 (Tyr14) (Rabbit, polyclonal) | Cell Signaling | Cat# 3251 RRID:AB_1002468 | IF (1:140)  WB (1:500) |
| antibody | anti-phospho-Tyr (Mouse, monoclonal) | Merck Chemicals | Cat# 05-321 RRID:AB_309678 | WB (1:1000) |
| antibody | anti-IgG (Rabbit, polyclonal) | Cell Signaling | Cat# 2729 RRID:AB_591709 | IP (1:500) |
| antibody | anti-CD36 (Rabbit, monoclonal) | Abcam | Cat# ab133625  RRID:AB_2716564 | WB (1:1000) |
| antibody | anti-Vinculin (Mouse, monoclonal) | Sigma | Cat# V9131 RRID:AB_477629 | IF (1:100)  WB (1:200) |
| antibody | anti-GM130 (Mouse, monoclonal) | BD Transduction Lab | Cat# 610822 RRID:AB_610822 | IF (1:100) |
| antibody | anti-Cdh5 (Goat, polyclonal) | R&D Systems | Cat# AF938 RRID:AB_355726 | IF (1:70) |
| antibody | anti-Cdh5 (Rabbit, monoclonal) | Cell Signaling | Cat# 2500 RRID:AB_10839118 | IF (1:100) |
| antibody | anti-Cdh5 (Mouse, monoclonal) | Santa Cruz | Cat# sc-9989 RRID:AB_2077957 | IF (1:100) |
| antibody | anti-Golph4 (Rabbit, polyclonal) | Abcam | Cat# ab28049 RRID:AB_732692 | IF (1:500) |
| antibody | anti-CD36 (Mouse, monoclonal) | Becton Dickinson | Cat# 552544 RRID:AB_2072646 | IF (1:100) |
| antibody | Alexa Fluor™ 647 Phalloidin | Invitrogen | Cat# A22287 RRID:AB_2620155 | IF (1:100) |
| antibody | anti-chicken Alexa Fluor-488 (Goat, polyclonal) | Jackson Immuno Research | Cat# 103-545-155  RRID:AB_2337390 | IF (1:500) |
| antibody | anti-rabbit Alexa Fluor-488 (Donkey, polyclonal) | Invitrogen | Cat# A21206 RRID:AB_141708 | IF (1:500) |
| antibody | anti-rat Cy3 (Donkey, polyclonal) | Jackson Immuno Research | Cat# 712-165-150 RRID:AB_2340666 | IF (1:100) |
| antibody | anti-rabbit Alexa Fluor-546 (Donkey, polyclonal) | Invitrogen | Cat# A10040 RRID:AB_2534016 | IF (1:500) |
| antibody | anti-goat Alexa Fluor-488 (Donkey, polyclonal) | Invitrogen | Cat# A11055 RRID:AB_2534102 | IF (1:500) |
| antibody | anti-goat Alexa Fluor-568 (Donkey, polyclonal) | Invitrogen | Cat# A11057 RRID:AB_142581 | IF (1:500) |
| antibody | anti-rat HRP-linked (Donkey, polyclonal) | Jackson Immuno Research | Cat# 712-035-153 RRID:AB_2340639 | IF (1:100) |
| antibody | anti-rabbit IgG HRP-linked (Goat) | Cell Signaling | Cat# 7074 RRID:AB_2099233 | WB (1:15000) |
| antibody | anti-mouse IgG 656G HRP-linked (Sheep) | GE-Healtcare | Cat# NA931 RRID:AB_772210) | WB (1:40000) |
| antibody | anti-goat IgG (H+L) Peroxidase AffiniPure Bovine | Jackson Immuno Research | Cat# 805-035-180 RRID:AB_2340874 | WB (1:15000) |
| antibody | anti-goat Alexa Fluor-488 (Donkey, polyclonal) | Invitrogen | Cat# A11055 RRID:AB_2534102 | IF (1:500) |
| antibody | anti-rat Alexa Fluor-647 (Donkey, polyclonal) | Jackson Immuno Research | Cat# 712-605-153 RRID:AB_2340694 | IF (1:500) |
| antibody | Streptavidin Alexa Fluor-488 | Invitrogen | Cat# S11223 | IF (1:100) |
| antibody | Streptavidin Alexa Fluor-647 | Invitrogen | Cat# S32357 | IF (1:100) |
| antibody | anti-human IgG (Fc specific) (Goat, polyclonal) | Jackson Immuno Research | Cat# 109-005-098 RRID:AB_2337541 | 10µg/ml |
| sequence-based reagent | Human GAPD (GAPDH) Endogenous Control (VIC™/MGB probe, primer limited) | Applied Biosystems | Cat# 4326317E | TaqMan probe Hs99999905_m1 |
| sequence-based reagent | Human EPHB4 TaqMan™ Gene Expression Assay (FAM) | Applied Biosystems | Cat# 4331182 | TaqMan probe Hs00174752_m1 |
| sequence-based reagent | Human CAV1 TaqMan™ Gene Expression Assay (FAM) | Applied Biosystems | Cat# 4331182 | TaqMan probe Hs00971716_m1 |
| sequence-based reagent | Human CD36 TaqMan™ Gene Expression Assay (FAM) | Applied Biosystems | Cat# 4331182 | TaqMan probe Hs00354519_m1 |
| peptide, recombinant protein | Recombinant Human IgG1 Fc protein | R&D Systems | Cat# 110-HG |  |
| peptide, recombinant protein | Recombinant Human Ephrin-B2 Fc | Biotechne | Cat# 7397-EB |  |
| peptide, recombinant protein | Recombinant Mouse EphB4 Fc | R&D Systems | Cat# 466-B4 |  |
| commercial assay or kit | TSA® Plus Cyanine 3 (Cy3) detection kit | Perkin Elmer | Cat# NEL744 |  |
| commercial assay or kit | BCA Protein Assay Kit | Pierce | Cat# 23225 |  |
| commercial assay or kit | RNeasy Mini Kit | QIAGEN | Cat# 74104 |  |
| commercial assay or kit | iScript cDNA Synthesis Kit | BIO-RAD | Cat# 170-8891 |  |
| commercial assay or kit | SsoAdvanced Universal Probes Supermix | BIO-RAD | Cat# 172-5284 |  |
| commercial assay or kit | Hypoxiprobe Plus kit-FITC | Hypoxiprobe | Cat# HP2 |  |
| commercial assay or kit | Cell Death Detection Kit TMR red | Roche | Cat# 12156792910 |  |
| commercial assay or kit | TrueSeq Stranded RNA LT Kit Ribo-Zero Gold | Illumina | Cat# 15032619 |  |
| commercial assay or kit | Pierce™ Coomassie Plus (Bradford) Assay Kit | Thermo Scientific | Cat# 23236 |  |
| commercial assay or kit | Precision Red Advanced protein assay | Cytoskeleton | Cat# ADV02 |  |
| chemical compound, drug | PP2 | Tocris | Cat# 1407 | final concentration: 25µM |
| chemical compound, drug | LY294002 | Tocris | Cat# 1130 | final concentration: 25µM |
| chemical compound, drug | U0126 | Promega | Cat# V1121 | final concentration: 10µM |
| chemical compound, drug | Sucrose | Sigma | Cat# S0389 |  |
| chemical compound, drug | cOmplete ULTRA Tablets Protease Inhibitor Cocktail | Roche | Cat# 05892970001 |  |
| chemical compound, drug | phosphatase inhibitor cocktail set V | EMD Millipore | Cat# 524629 |  |
| chemical compound, drug | Gelatine | Sigma | Cat# G1890 |  |
| chemical compound, drug | Polyvinylpyrrolidone | Sigma | Cat# P5288 |  |
| chemical compound, drug | Albumin from Bovine Serum (BSA), Alexa Fluor™ 555 conjugate | Molecular Probes | Cat# A34786 | final concentration: 4µM |
| chemical compound, drug | Transferrin From Human Serum, Alexa Fluor™ 488 Conjugate | Invitrogen | Cat# T13342 | final concentration: 50µg/ml |
| chemical compound, drug | BODIPY™ 500/510 C1, C12 | Molecular Probes | Cat# 3823 | final concentration: 5µM |
| chemical compound, drug | Fatty-acids free BSA | Sigma | Cat# A9205 |  |
| chemical compound, drug | Insulin solution human | Sigma | Cat# I9278 |  |
| chemical compound, drug | Trypsin-EDTA solution | Sigma | Cat# T3924 |  |
| chemical compound, drug | Paraformaldehyde | Sigma | Cat# P6148 |  |
| chemical compound, drug | Fluoromount-G | Southern Biotech | Cat# 0100-01 |  |
| chemical compound, drug | ECL™ Prime Western Blotting Detection Reagent | GE-Healthcare | Cat# RPN2236 |  |
| chemical compound, drug | 4-hydroxy tamoxifen | Sigma | Cat# H7904 |  |
| chemical compound, drug | Oil Red-O | Sigma | Cat# O0625 |  |
| chemical compound, drug | Hematoxilin | Sigma | Cat# MHS16 |  |
| chemical compound, drug | Dimethyl sulfoxide | Sigma | Cat# D8418 |  |
| chemical compound, drug | EBM-2 endothelial cells medium | Lonza | Cat# CC-3156 |  |
| chemical compound, drug | EGM-2 Single Quots | Lonza | Cat# CC-4176 |  |
| chemical compound, drug | HEPES | Sigma | Cat# H3537 |  |
| chemical compound, drug | Ketamine | Zoetis | Cat# 344771 |  |
| chemical compound, drug | Rompum | Bayer healthcare | Cat# D-51368 |  |
| software, algorithm | ImageJ (v2.0.0 Fiji) | Schindelin et al., 2012 | RRID:SCR_002285 |  |
| software, algorithm | Volocity (v6.3) | Perkin Elmer | RRID:SCR_002668 |  |
| software, algorithm | Illustrator (vCC2018) | Adobe | RRID:SCR_010279 |  |
| software, algorithm | GraphPad Prism7 | GraphPad Software | RRID:SCR_002798 |  |
| software, algorithm | FlowJo (v10.3) | BD Life Sciences | RRID:SCR_008520 |  |
| software, algorithm | Xcalibur software | Thermo Scientific | RRID:SCR_014593 |  |
| software, algorithm | MaxQuant (v.1.6.2.6) | Cox and Mann, 2008 | RRID:SCR_014485 |  |
| software, algorithm | Perseus (v.1.6.2.1) | Tyanova et al., 2016 | RRID:SCR_015753 |  |
| software, algorithm | PUNIAS software | http://punias.free.fr/ |  |  |
| other | DAPI stain | Sigma | Cat# D9542 | (1mg/ml) |
